# Spatiotemporal Prediction of Increasing Winter Perceived Temperature across a Sub-Tropical City for Sustainable Planning and Climate Change Mitigation

**DOI:** 10.3390/ijerph16030497

**Published:** 2019-02-11

**Authors:** Hung Chak Ho, Sawaid Abbas, Jinxin Yang, Rui Zhu, Man Sing Wong

**Affiliations:** 1 Department of Urban Planning and Design, The University of Hong Kong, Hong Kong; 2 Department of Land Surveying and Geo-Informatics, The Hong Kong Polytechnic University, Kowloon, Hong Kong; sawaid.abbas@gmail.com; 3 School of Geographic Sciences, Guangzhou University, 510000 Guangzhou, China; jinxin.yang@connect.polyu.hk; 4 Senseable City Laboratory, Singapore-MIT Alliance for Research and Technology, Singapore; rui.zhu@smart.mit.edu; 5 Research Institute for Sustainable Urban Development, The Hong Kong Polytechnic University, Hong Kong

**Keywords:** climate change, community design, socioeconomic deprivation, sustainable planning, urban morphology

## Abstract

Climate variability has been documented as being key to influencing human wellbeing across cities as it is linked to mortality and illness due to changes in the perceived weather cycle. Many studies have investigated the impact of summer temperature on human health and have proposed mitigation strategies for summer heat waves. However, sub-tropical cities are still experiencing winter temperature variations. Increasing winter perceived temperature through the decades may soon affect city wellbeing, due to a larger temperature change between normal winter days and extreme cold events, which may cause higher health risk due to lack of adaptation and self-preparedness. Therefore, winter perceived temperature should also be considered and integrated in urban sustainable planning. This study has integrated the increasing winter perceived temperature as a factor for developing spatiotemporal protocols for mitigating the adverse impact of climate change. Land surface temperature (LST) derived from satellite images and building data extracted from aerial photographs were used to simulate the adjusted wind chill equivalent temperature (AWCET) particularly for sub-tropical scenarios between 1990 and 2010 of the Kowloon Peninsula, Hong Kong. Compared with perceived temperature based on the representative station located at the headquarters of the Hong Kong Observatory, the temperature of half the study area in the Kowloon Peninsula has raised by 1.5 °C. The areas with less green space and less public open space in 2010 show higher relative temperatures. Socioeconomically deprived areas (e.g., areas with lower median monthly income) may suffer more from this scenario, but not all types of socioeconomic disparities are associated with poor sustainable planning. Based on our results and the “no-one left behind” guideline from the United Nations, climate change mitigation should be conducted by targeting socioeconomic neighborhoods more than just aging communities.

## 1. Introduction

Thermal distress is an issue contributing to negative wellbeing across cities [1]. Numerous studies have indicated that perceived temperature is a key part of inducing thermal distress and is partially controlled by urban built environments [2]. During a heat wave, the urban population in a city with a poorly designed built environment can suffer intensive thermal distress, and this thermal distress may even lead to thermal health issues in extreme cases [3]. Therefore, prediction of perceived temperature for monitoring thermal distress is the key to the wellbeing of urban populations in climate change mitigation [4].

Researchers have developed indices from apparent temperature to represent perceived temperature. For example, humidex and the heat stress index have been applied to describe thermal distress from citizens during extreme weather events [5,6,7,8,9]. In addition, a spatial pattern of perceived temperature has also been estimated using these indices [6]. However, the data used for such studies were always acquired by mobile or vehicular transverse observations. Further improvements in mapping perceived temperature have been undertaken by incorporating satellite images for spatial and temporal monitoring of thermal stress. Satellite-based predicted maps are highly accurate and can provide a synoptic view of a city without extensive field measurements [7,8,9]. Moreover, climate resilience research also included a satellite-based perceived temperature map as a fundamental variable for developing guidelines to reduce summer thermal discomfort [10,11].

Under the influence of climate change, global temperature will rapidly increase in the coming decades [12,13]. This may result in a decreased of number of normal winter days, especially in cities within the sub-tropical region. However, there have been studies indicating that global warming cannot minimize the adverse health effect of extremely cold days, and high mortality/morbidity risks can still be observed during extremely cold days in sub-tropical cities [14]. This is because the health burden of extreme cold events is mainly from sudden decreases in temperature. In a city with more relatively hot days during winter, an extreme cold event can be a fatal disaster due to lack of adaptation and self-preparedness of local people. Therefore, it is necessary to develop further planning for climate change mitigation for local populations. Specifically, the key for the mitigation of climate change should include the improvement of the urban resilience of a city [15], and the way to do this is by the further enhancement of sustainable planning. Based on this view, the strategy of mitigating such extreme weather should be based on normalizing the temperature across a sub-tropical city to a winter-like scenario, in order to develop a nature-based solution that can improve climate change adaptation of local population.

In order to better develop a comprehensive protocol for sustainable planning and for climate change mitigation in cities with less normal winter days, one solution will be an integration of winter perceived temperature in the prediction modelling. Particularly, a spatiotemporal component that can represent the increase of winter temperature through the years should be incorporated, since this strategy can improve planning guidelines to reduce human thermal discomfort in all-seasons under the impact of climate change. It is also essential to pinpoint an urban area with inner-city decay, in which the planning protocols can be implemented as sustainable development during urban renewal. In addition, such planning protocols should also be incorporated with socioeconomic data for modeling. There are also extensive studies that aim to identify socioeconomically deprived neighborhoods for planning, and these have worked with temperature maps as well as with the data on socioeconomic deprivation in order to identify the risk spots for climate change mitigation [2,3,10].

Based on these hypotheses, here we present a study which provides a first attempt of using spatiotemporal information of winter perceived temperature for improving the urban design for the climate resilience of a subtropical city. The specific objectives of this study are: (1) to estimate the average increase in winter perceived temperature in five-year intervals based on 20–year data between 1990 and 2010; (2) to compare the areas with an increase/decrease in winter perceived temperatures with urban environmental factors of the same locations; (3) to pinpoint the hot-spot areas which require immediate improvements in urban design; (4) to further pinpoint the hot-spot areas based on the identification of different socioeconomic deprived neighborhoods; (5) and to highlight key factors for future urban planning. A case study for planning application was applied to the Kowloon Peninsula of Hong Kong, a well-known high-density environment in a compact city with mixed land use and a large population across a relatively small geographic extent [16].

## 2. Study Area

Kowloon Peninsula of Hong Kong is an urban environment with mixed land use and high-density settings. In a small area of only 26 km^2^ bounded by mountains in the north and the harbor surrounding the peninsula, there were approximately 1 million people based on the 2011 Census report [16]. Due to a history of development, despite the small area and large local population, the Kowloon Peninsula has a high percentage of commercial areas, including town centers and commercial landmarks of Hong Kong, such as Tsim Sha Tsui and Mong Kok. Based on datasets delineated from the 1990 coastal boundary, 28.1% of our study area was residential or commercial lands, 13.4% was government, institutional and community facilities, while only 8.9% was public open space including public parks recognized by the 2009 land utilization map from the Planning Department of Hong Kong. Furthermore, due to continuing development and re-development across the Kowloon Peninsula, a notable number of high-rise buildings were observed across the whole region. Based on a set of 2010 building GIS data, the average building height of the study area (excluding lands from reclamation after 1990) was 26.8 m, with a standard deviation of height of 14.0 m, while the highest one in 2010 reached 87.8 m. This urban morphology has formed a complex environment that can influence the microclimate, for example, with urban canyons that affect both ventilation and air temperature [17,18,19,20]. Significantly high temperatures and relatively low wind speeds were therefore widely observed across the high-density environment in summer. However, despite such factors being associated with thermal distress in hot summer days [21], previous studies have documented that cold temperatures can contribute to health risks in local people [22], due to the sudden decrease in temperature. In addition, cold spells (e.g., 2008 Chinese winter storms; the January 2016 East Asia cold wave) are expected to increase through climate change in Hong Kong. Based on records retrieved from the weather station located at the Hong Kong Observatory (which is also within urbanized built-up areas of Hong Kong and within our study area), the average temperature of December 2010 was 2.7 °C higher than the average temperature of December 1910, and 1.2 °C higher than temperature of December 1960. Such increasing temperatures can result in a significant drop in temperature during an extreme cold event that can contribute to higher mortality or morbidity risk. Therefore, it is necessary to include winter perceived temperature in modelling to develop better protocols of sustainable urban development.

## 3. Methods

### 3.1. Data Collection

**Figure 1:**
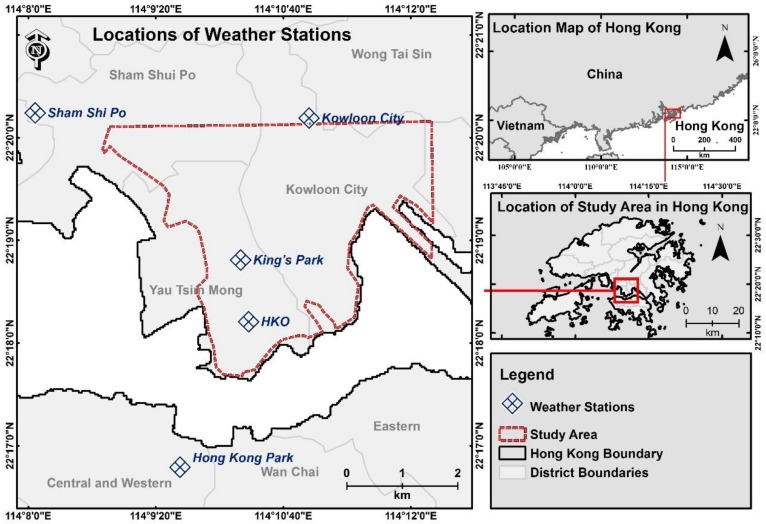
Study site. Red dotted lines are the boundary of the study area. Blue symbols are the weather stations within or close to the study area.

Hourly temperature data from weather stations were obtained from the Hong Kong Observatory, including five stations located in Sham Shui Po, Kowloon City, King’s Park, the Hong Kong Observatory Headquarters and Hong Kong Park (Figure 1). These weather stations are the government-based weather stations located within or near the study area. These stations were representatives of the urban environments of Hong Kong, and were suitable for mapping air temperatures across the high-environment environments of the Kowloon Peninsula. In addition, wind information for modelling was obtained from the Star Ferry Automatic Wind Station in the southwestern corner of Peninsula and wind data for validation was obtained from the weather station located at the King’s Park and the Hong Kong Observatory Headquarter.

Twenty-three Landsat 5 multispectral images between 1990 and 2011 were obtained from the USGS Global Visualization Viewer (GloVis), including 18 images between 2006 and 2011, as training data for air temperature prediction, and 5 additional images as representatives of typical winter days during 1990, 1995, 2000, 2005, and 2010 (Table 1 and Table 2). These data were selected due to their availability and lack of cloud contamination over the study area. A series of digital surface model (DSM) with 2 m spatial resolution and three-dimensional (3D) building datasets were retrieved from stereoscopic photogrammetry of aerial photographs for years 1990, 1995, 2000, 2005, 2010 [16]. These DSMs were used to represent the urban morphology of Kowloon Peninsula and for mapping the ventilation across the urban environment.

This study also obtained district-level demographic data, vegetation maps, land use information, and sky view factor (SVF) datasets for comparative analyses. Demographic data were retrieved from the 2011 Hong Kong Census in a the “Tertiary Planning Unit”, a small unit for regional planning purposes in Hong Kong. Vegetation cover was mapped based on land information from the Hong Kong Planning Department in 10 m spatial resolution. Land use information was retrieved from a 2009 land utilization dataset mapped by the Hong Kong Planning Department with 27 classes at 10 m resolution. SVF was modeled based on the 3-dimensional building information of Hong Kong retrieved from 2011 LiDAR data, with methods documented in a previous study [23].

### 3.2. Estimation of Adjusted Wind Chill Equivalent Temperature (AWCET)

In this study, an adjusted wind chill equivalent temperature (AWCET) was developed and used to represent the winter perceived temperature of a high-density environment [24]. In brief, the original new wind chill equivalent temperature (NWCET) was calculated by air temperature and wind velocity based on the following Equation:

$$NWCET=13.12+0.6215\times Ta-11.37\;WV^{0.16}+0.3965\times Ta\times \;WV^{0.16}$$

where *Ta* is the air temperature in °C and *WV* is the wind velocity in km/hour.

This original NWCET was designated for a cold scenario [5] and was evaluated to be relevant to human discomfort and health risk during wintertime [25]. This index is also used as a governmental measure of winter perceived temperature in the United States and Canada [24]. However, since NWCET is designed for estimating perceived temperature during a cold season in temperate regions, this study has modified the index to an AWCET that is more suitable to sub-tropical scenario. After tuning the index in this study, it was able to evaluate the difference in temperature through the years for further understanding of how an extreme cold event in a sub-tropical city with an increasing temperature can be more serious than for areas with a “typical winter” (e.g., Europe, United States). In detail, the most arguable part of the original NWCET is the potential overestimation of wind chill effect on human health in subtropical regions. Therefore, we adjusted the index by reducing the wind load to be 3 to 4 times weaker than the original index as follows:

$$AWCET=13.12+0.6215\times Ta-11.37\;{\left(\frac{WV}{3.6}\right)}^{0.16}+0.3965\times Ta\times {\left(\frac{WV}{3.6}\right)}^{0.16}$$

where *Ta* is the air temperature in °C and *WV* is the wind velocity in km/hour.

This AWCET was further evaluated by mortality data from between 2008 and 2012 in Hong Kong, based on four sets of time-stratified analyses. Based on the analyses, AWCET have significantly higher mortality in days with lower temperatures of Hong Kong. It indicated that an adjusted index is useful in sub-tropical cities.

In order to estimate the AWCET in this study, spatial maps of air temperature and wind velocity for each year of study (1990, 1995, 2000, 2005, 2010) were prepared as follows.

The spatial distribution of air temperature of each year was estimated based on the land surface temperature (LST) dataset derived from Landsat 5 images and air temperature records obtained from all the government-based weather stations stated above. Details of the LST retrieval used in this study can be referred to in reference [26]. In brief, this is an advanced urban exitance model designated for retrieving LST with a relatively high spatial resolution (30 m) in a compact city, with considerations of the urban geometric effects and material emissivity. Although some research has shown complex relationships between LST and air temperature [7,27], a previous study in Hong Kong found that using linear regression for the Kowloon Peninsula is still appropriate [28], as this area is mostly urbanized and relatively flat. Lower spatial variation across urban areas has less influence on the temporal variation of daily air temperature, similar to other studies that have used LST images with linear regression to predict the daily average temperature in a spatially homogenous area. Therefore, eighteen Landsat 5 images acquired in the winters between 2006 and 2011, and the daily average of air temperature records on the overpass date of satellites, were firstly used to develop a training set of the prediction model. LST datasets selected as representative of the year were then fitted into the prediction model. It is noted that the selected LST dataset for 2010 was not included in the training data and was used for validation purposes instead. The deliverables were five different maps at 30 m resolution showing air temperature distribution across Kowloon Peninsula in the year of 1990, 1995, 2000, 2005, and 2010. These results were then compared with the daily average air temperature retrieved from the five weather stations on the same day for validation. Root-mean-square error (RMSE) and R-Squared (R^2^) were used to determine the accuracy of air temperature prediction by LST products.

Wind velocity across the Kowloon Peninsula was estimated based on the Airflow Analyst^®^, as an extension plugin of ArcGIS. This model can incorporate spatial data for simulating ventilation and air movements across a complex landscape. The algorithm was based on a 3-dimensional computed fluid dynamics (CFD) model, and has been validated in various studies for predicting wind speed in different urban and rural environments [29,30,31]. In this study, the DEM and 3D building datasets for 1990, 1995, 2000, 2005, and 2010 were inputted to simulate the wind velocity across the Kowloon Peninsula. Based on the daily average of wind information retrieved from the Star Ferry Automatic Wind Station on the representative dates of each study year, five sets of data corresponding to each 3D scenario of each study year were generated. These 3D scenarios were then applied to subsequent fluid analysis at an average surface height of 1.5 m. Results were exported as 2D raster maps of each year at 10 m spatial resolution for illustrating wind velocity under the influence of urban sprawl and morphology.

For estimating the AWCET, air temperature maps were resampled to 10 m resolution with the same resolution as wind velocity data. Based on the resampled air temperature dataset and wind velocity map of each year, this study applied the equation stated above to map the AWCET across the Kowloon Peninsula for the year of 1990, 1995, 2000, 2005, and 2010.

### 3.3. Comparative Analyses of Increasing Winter Perceived Temperature and Urban Environment

In order to conduct a comparative assessment, each perceived temperature map was subtracted by the AWCET at the location of the Hong Kong Observatory Headquarters for mapping the relative AWCET across the Kowloon Peninsula each year. The aim here was to evaluate the intensity of heat across districts based on a representative station and the method is commonly used in urban climatic studies [7,16]. These relative AWCET maps were further processed as follows: (1) we subtracted the relative AWCET maps of 1995, 2000, 2005 and 2010 by the winter perceived temperature maps demonstrating scenarios from past 5 years (e.g., a map of 1995 was subtracted by a map of 1990); and (2) we averaged all the relative subtracted maps for estimating the average increase of relative AWCET across Kowloon Peninsula. The resulting maps were then integrated with information on the urban environment: (1) for indicating whether current urban settings can induce/mitigate the potential increase of winter temperature, and (2) for identifying the community groups (e.g., senior populations, low income) that may suffer more thermal distress under the changing environment. The average relative AWCET for 20 years (1990–2010) was also mapped for evaluating whether spatial distributions between average AWCET and increase of AWCET are different. It was based on an assumption that the spatial distribution of average temperature commonly used for climatic mapping was significantly different from the distribution of the increase in AWCET, and therefore the latter one should be applied for sustainable urban planning in order to develop a set of solutions for climate change mitigation. This study also mapped both distributions based on the lands within the 1990 coastal boundary of Hong Kong, for a better and more accurate comparison of temperature changes within 20 years. Lands from reclamation after 1990 were not included in this analysis.

For the evaluation of the relationship between current urban environments and increased winter perceived temperature, we further converted the map of average relative increases of AWCET to a vector-based point format in order to represent information of each location at a 30 m resolution across Kowloon. Four groups of variables were created for data comparison: (1) green coverage; (2) public open space; (3) road network, and (4) building density, in which more of items (1) and (2) and less of (3) and (4) have been identified as keys for climate change mitigation through urban planning [32,33]. In brief, data of green coverage were derived from the vegetation map based on the focal statistics function of ArcGIS, and the derived data indicated the percentage of green coverage within radii of 100 m, 200 m, 300 m, 400 m, and 500 m, respectively. Public open spaces and road networks were retrieved from the “open space” and “road” GIS-based land use data, and these data subsets were also spatially averaged by focal statistics for estimating the percentage of public open spaces or road networks within a radii of 100 m, 200 m, 300 m, 400 m, and 500 m. Building density was demonstrated by SVF, where higher SVF indicated lower building density and lower SVF indicated higher building density [34,35]. These data were also spatially averaged by focal statistics as stated above. All data for comparison were extracted by all points across the study area in representing the environmental factors at 30 m interval.

*T*-test analysis was applied to compare the difference in the mean of each urban environmental factor for areas with an increase of AWCET (>0 °C) and areas without an increase (<=0 °C) through the years. The *T*-test was firstly applied to compare the areas with an increase or without an increase of AWCET across the study site was applied to stratified areas with higher socioeconomic deprivation for further evaluation of the difference in the mean of areas with and without an increase of AWCET across the socioeconomically deprived area. The following are the socioeconomically deprived areas that have been analyzed in this study: (1) areas with higher percentage of senior population (age >=65); (2) areas with higher percentage of children and young adolescent populations (age <=14); (3) areas with lower median monthly income from main employment of working population, excluding unpaid family workers, and (4) areas with lower percentage of labor force participation rate. In detail, the medians of all tertiary planning units (TPUs) for the socioeconomic factors stated above were used as the cutoff for stratifying districts under the categories of (1) to (4).

## 4. Results

### 4.1. Spatial Datasets for Adjusted Wind Chill Equivalent Temperature (AWCET) Estimation

**Figure 2:**
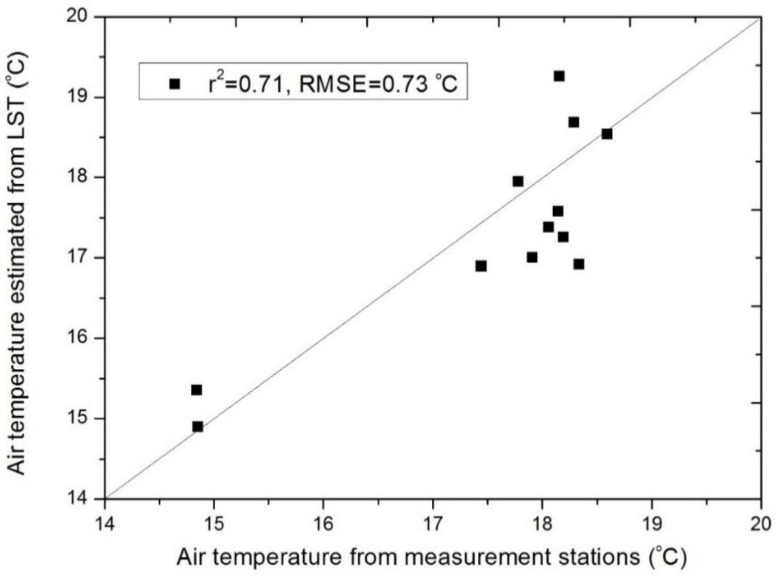
Comparison between predicted and observed data. Y-axis represents the values of predicted data from the regression in this study. X-axis represents the values of observed data from weather stations.

**Figure 3:**
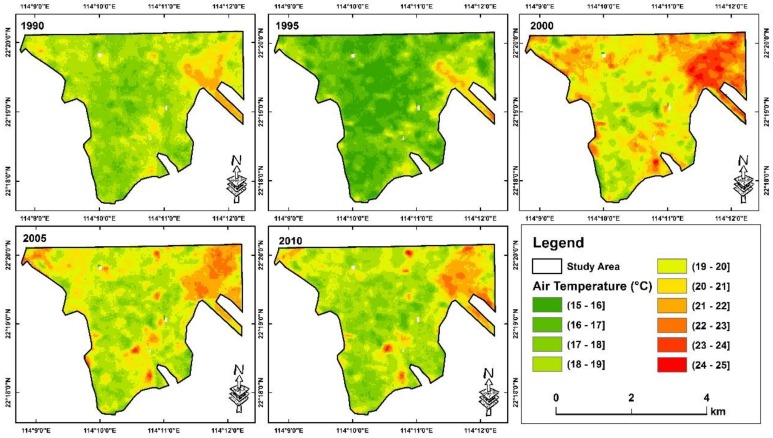
Predicted air temperature across study area in 1990, 1995, 2000, 2005 and 2010.

**Figure 4:**
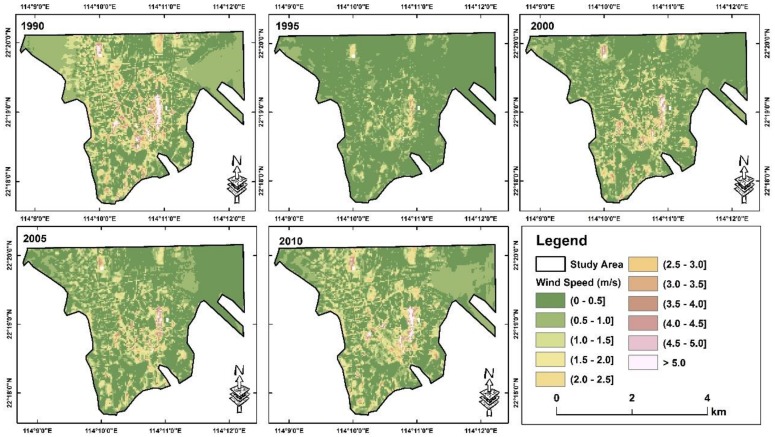
Predicted wind speed across study area in 1990, 1995, 2000, 2005 and 2010.

By fitting the training dataset of air temperature into a linear regression, predicted air temperature on the selected date with satellite overpass of the study year have shown a high correlation with the observed air temperature on the same day, in which RMSE is 0.73 °C and R^2^ is 0.71 (Figure 2). These results are comparable with other similar studies, which RMSEs of previous studies can range from 1.3–3.5 °C, and R-squared ranged from 0.34 to 0.94 [7,27,28,36,37]. Based on the predicted maps (Figure 3), the air temperature across districts are apparently increasing, while the wind speed (Figure 4) is varying through years, possibly due to urban development and re-development, or regional impact. Therefore, it is necessary to combine both, mapping the relative increase of AWCET for determining the actual change in winter perceived temperature that potentially influences the city wellbeing.

### 4.2. Comparison of Relative AWCET Increase and Urban Environmental Factors

**Figure 5:**
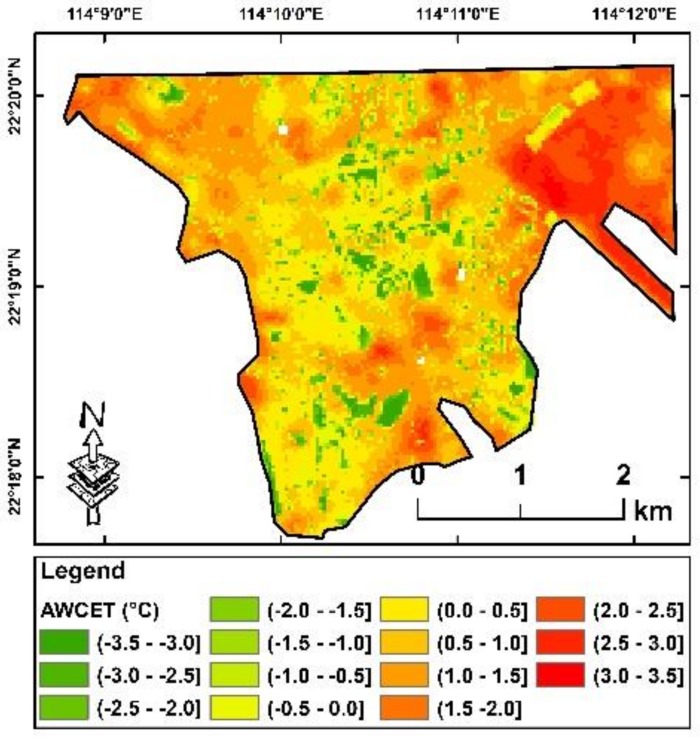
The average of relative AWCET between 1990 and 2010. Red indicates areas with higher relative AWCET and green indicates areas with lower relative AWCET.

**Figure 6:**
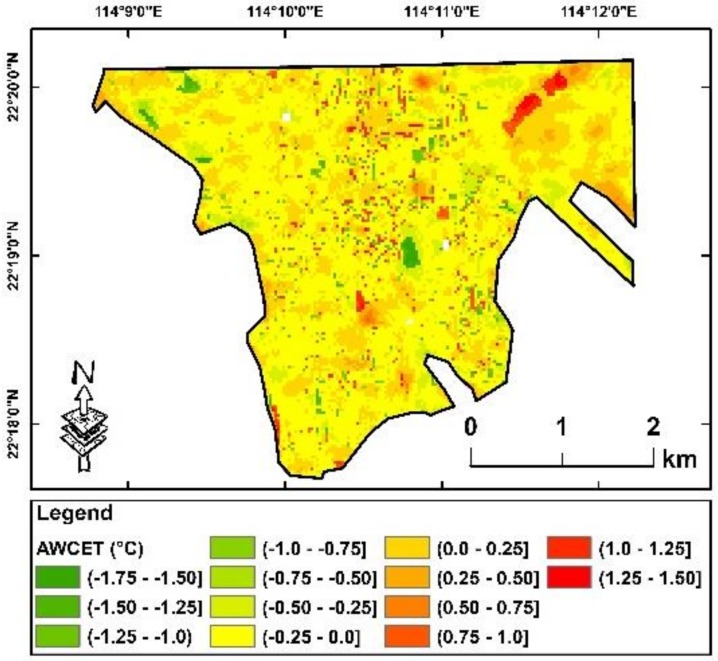
The average increase of relative AWCET of every five years between 1990 and 2010. Red indicates areas with higher increase of relative AWCET and green indicates areas without increase of relative AWCET.

Based on the results above, two resultant maps have been mapped as follows: (1) the average of relative AWCET between 1990 and 2010 (Figure 5), and (2) the average increase of relative AWCET every five years between 1990 and 2010 (Figure 6). Although about half of the regions in the study area have higher relative AWCET geographically, approximately half of the regions of this area of interest also have a higher than average increase of relative AWCET. These two subsets of “high-areas” were not spatially overlapped. A further comparison between these two maps was conducted, in which there was no correlation between the two maps (R^2^ = 0.002). It implied that only using temperature across districts for climate change mitigation is inadequate, and that reduction of adverse impacts of climate change should include the increase of perceived temperature, especially increases in winter temperature.

By overviewing the urban environment across the Kowloon Peninsula, this study has found that areas with an increased relative AWCET were surrounded by areas in 2010 with a lower percentage of urban green coverage and public open space than the areas without an increase of relative AWCET. The *T*-tests (Table 3) have shown that the areas with an increase of AWCET had significantly (1.9%, 1.0%, 0.8%, 0.6%, and 0.7%) lower green coverage across spatial buffers of 100 m, 200 m, 300 m, 400 m, and 500 m radii, on average. Although there was no significant difference in the percentage of public open space between areas with and without increase of relative AWCET for the spatial buffers of 100 m and 200 m, this study also analyzed larger spatial extents (radii of 300 m, 400 m and 500 m). The areas with increased relative AWCET still had a 0.8–0.9% lower percentage of public open space than the areas without an increase. In contrast, the percentages of road network and average building density were not higher in the areas with an AWCET increase. This implies that the current urban settings have already been incorporated in reducing building density and road networks, however, sustainable design combined with urban greenery and public open space may still be insufficient, especially in targeting climate change mitigation.

### 4.3. AWCET Increases and Urban Environments across Socioeconomically Deprived Areas

**Figure 7:**
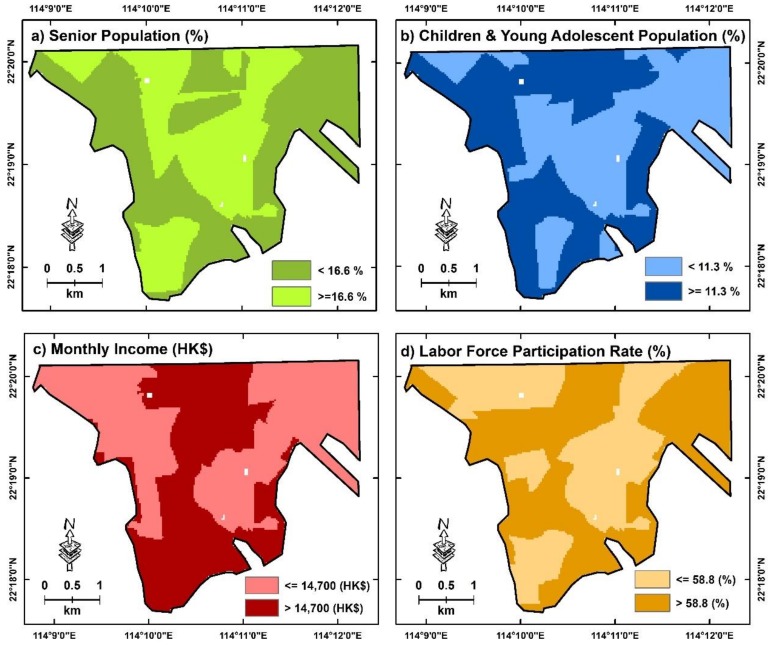
Areas with higher and lower socioeconomic deprivation based on different demographic factors retrieved by the 2010 census of Hong Kong.

Areas of interest in this study have covered 45 TPUs across the Kowloon Peninsula, Hong Kong. Among these 45 TPUs, the percentage of senior population in 2010 ranged from 3.1% to 26.0%, with a median of 16.6%. The percentage of children and young adolescent population ranged from 6.4% to 16.5% with a median of 11.3%. In addition, the monthly income from the main employment of the working population, excluding unpaid family workers, ranged from HKD$9,000 to HKD$50,000 with a median of HKD$14,700. Additionally, the labor force participation rate ranged from 48.7% and 71.9% with a median of 58.8%. Based on the median of all factors, we assigned TPUs with a percentage of senior population of >=16.6% or a percentage of children and young adolescent population of >= 11.3%, or a monthly income from main employment of <=$14,700, or a labor force participation rate of <= 58.8% in socioeconomically deprived neighborhoods, namely in “areas with a higher senior population”, “areas with a higher children and young adolescent population”, “areas with a higher lower income population”, and “areas with lower labor participation rate” (Figure 7).

Based on a stratification of identified neighborhoods with higher socioeconomic deprivation, significant disparities in urban environments across different neighborhoods were observed. In brief, areas with higher low-income populations had much less greenspace and public open space than other areas across the Kowloon Peninsula, while areas with a higher senior population, which are usually identified as vulnerable neighborhoods due to aging issues in a city, had a much higher percentage of greenspace and public open space than other areas. These results also led to further disparities when areas with or without an increase in AWCET were compared and evaluated using the *T*-test. For areas with a higher senior population (Table 4), this study observed that the need for more greenspace in these areas was similar to that in areas with an increase in AWCET. This is because the only group with a significantly lower mean was the area with an increase in AWCET and a lower percentage of vegetation within a 100 m radius. More areas have emerged with protective factors in recent years, especially areas with higher populations of children and young adolescent with increases in AWCET. Compared with the areas without increases in AWCET, we have only found a significantly lower mean percentage of public open space estimated based on a 500 m radius in areas where increases in AWCET exist, while this has not been found with the use of a radius between 100 m and 400 m.

In contrast to areas with a higher senior population or areas with children and young adolescent populations that had more protective areas for reducing thermal distress, areas with high low-income populations were expected to suffer from urban thermal issues. In addition to those areas that already had less greenspace and public open space than other types of areas, the areas with an increase in AWCET had an average of between 2.1% and 4.0% less greenspace with a radius of 100 m to 500 m, compared to areas without an increase in AWCET. Among those areas with a higher low education population, the areas with an increase in AWCET had an average of between 1.5% and 3% less public open space in a radius of 100 m to 500 m. These results indicated that such neighborhoods will face severe effects of thermal stress on vulnerable populations due to the raising of temperature by climate change.

Finally, although areas with a lower labor participation rate had more greenspace and public open space than the general environment across Kowloon, the areas which increased in AWCET across these neighborhoods had less greenspace and public open space than the areas without increases in AWCET. It indicated that although such neighborhoods may be in good environmental quality, when extreme weather caused by climate change is more severe and frequent, less mitigation effort on areas with an increase in AWCET may ultimately result in thermal health issues for populations. In addition, higher percentages of road networks and average building density were not found in all socioeconomically deprived areas with increases in AWCET. This aligns with the results in the previous section, which stated that the actions of urban design might be applied across the city to reduce building density and road networks, in order to ultimately reduce thermal distress across the city.

## 5. Discussion

### 5.1. Implications and Guidelines for Sustainable Development

Based on the analysis, areas with significant increases in winter perceived temperatures were observed across the urban environments of a compact city. The results of this study have also indicated that urban design has been applied to the city for climate change mitigation. For example, areas with higher building density and a higher percentage of road network in recent years were not associated with areas of increased perceived temperature. However, there is still a need to improve the greenspace and public open space for mitigation across the urban environment; although the need for improvement is varied across neighborhoods, even among districts with different groups of vulnerable populations.

Based on these findings, a guideline for sustainable development should be established. First, a prediction of spatiotemporal change in winter perceived temperature for mitigation is a critical process because it is a key factor that has not been integrated with previous/existing protocols. Second, urban greenery and public open space should be increased across the city. While integrating these two factors, there are items that should be investigated further. For example, previous studies found that wind ventilation is the key to reducing thermal distress [16]. For the design of public open space to reduce the impact of increasing winter perceived temperatures, ventilation should be considered in order to increase the potential air flow across a compact city. In addition, since a compact city is a high-density environment with a large population, there are always limitations to further decreasing building density and road networks to increase the ratio of greenery or public open space. In this case, vertical greenery, green roofs and terrace gardens are important for increasing green coverage. In order for targeted improvement of the urban green environment to take place, two items are necessary: (1) avoid using artificial green roofs in a high-density city, and (2) use an appropriate selection of construction materials for the development of an open space. Unlike urban vegetation, artificial green roofs cannot provide evapotranspiration and thus they cannot reduce urban heat in a high-density environment. A previous study also found that inappropriate selection of construction materials for open spaces cannot passively reduce heat intensity across the space [38]. Moreover, additional care should be undertaken when selecting appropriate vegetative species for urban greenery. The species should be thoroughly considered keeping in mind their ability to reduce air pollution across the city, since a significant association between air pollution and thermal distress has been widely observed [39].

In addition to the selection of environmental criteria for sustainable urban improvement, understanding socioeconomic disparities when community planning is also important. While city planning for better environmental health should follow a scheme that can reduce issues caused by active aging, such as the “Towards an Age-Friendly World” scheme promoted by the World Health Organization (WHO), major problems in the socio-environment may be far more than just an issue of a large population of elderly people. For example, this study has found that the aging population has been well-protected in current urban design, while other types of vulnerable populations, such as lower income populations, have less protection from climate mitigation based on the current design of the city. Thus, planning for the improvement of urban environments should also focus on the needs of different vulnerable populations. This is also aligned with the sustainable development goal of the United Nations, in which “no-one left behind” is the core of future sustainable planning. Based on this, a multi-level urban design should be developed, in which macro-scale design for planning protocols of the general environment should be applied across a city and micro-scale planning with specific urban design should be conducted by weighting the proportion of vulnerable populations (e.g., the percentage of socioeconomically deprived populations).

### 5.2. Limitations and Future Directions

In this study, only government-based weather stations were used to map air temperature and for simulating the wind speed. However, recent studies have also pointed out that volunteered geographic information (VGI) may be able to enhance the urban climate prediction, especially in increasing spatial variability [7]. However, since the concept of VGI has only begun within the last decade [40], there is no relevant information for a longitudinal study. For future investigation, weather information from VGI networks, such as Weather Underground (www.wunderground.com) and the Community Weather Information Network (weather.ap.polyu.edu.hk), can be used to enhance spatial prediction of winter perceived temperature.

It is also important to note that this study has focused on developing sustainable protocols to mitigate climate change effects in cities facing fewer normal winter days. Such analysis is aimed at targeting environmentally vulnerable areas for improving urban resilience for all-season scenarios as long-term mitigation, which short-term strategies such as emergency planning might not provide. Thus, a future study to combine results of this study and disaster planning protocols for extremely cold days is essential for the purpose of developing a higher-hierarchy framework designed for city planning and land policy. The importance of our current results suggest that future study should be focused on the improvement of physical environments and urban design for long-term mitigation, instead of on disaster plans for extreme days that focus more on usage of the urban design for resource allocation, emergency response, and socioeconomic support [41].

Finally, this study used the AWCET instead of the temperature indices for all-seasons such as physiological equivalent temperature (PET) and universal thermal climate index (UTCI). It was based on a hypothesis that this study not only aimed to evaluate the discomfort caused by increasing temperature, but also to project a scenario of how the spatiotemporal variability of winter perceived temperature is different for the temperate city and how the magnitude of this difference is stronger when the relative AWCET has been increased. More importantly, previous study [42] has also noted that even common indices for all-seasons such as PET and UTCI are not interchangeable, since the design and representation of each index is totally different. Therefore, it is important to note that using different indices will significantly affect the results, while this must be based on hypotheses before modelling and applications.

## 6. Conclusions

Spatiotemporal variation in relative winter perceived temperatures across a high-density environment have been modeled, with a comparison of urban characteristics in different socioeconomically deprived neighborhoods for city planning. Results have shown that areas with increases in relative temperature may generally be the districts with less greenspace and public open space across high-density environments in recent years, but this can be varied in socioeconomically deprived districts. In addition, not all recognized factors of socioeconomic deprivation are associated with districts that have recent urban design with less efficiency for climate change mitigation. The results imply that further planning should have multi-level assessments of environmental impacts in order to develop protocols for climate change mitigations that can be both beneficial to the general population and to specific vulnerable populations.

## Figures and Tables

**Table 1:** Satellite images (Landsat 5 TM) for training regression for air temperature prediction.

Retrieved Dates of Landsat Images for Data Training of Air Temperature Prediction		
3 March 2011	2 February 2011	1 January 2011
29 October 2010	30 November 2010	18 September 2010
26 March 2010	14 January 2010	4 December 2009
3 February 2009	18 January 2009	2 January 2009
1 December 2008	17 December 2008	4 March 2008
29 January 2007	13 January 2007	28 December 2006

**Table 2:** Satellite images (Landsat 5 TM) for estimating average air temperature during the representative days.

Retrieved Dates of Landsat Images for Predicting Air Temperature at the Representative Dates (Same as the Retrieved Date)				
24 December 1990	30 December 1995	11 February 2000	23 January 2005	23 December 2010

**Table 3:** Comparison of the average increase in AWCET for every five years and areas with poorer/better sustainable planning in the current year in general environment. Bold text indicates results with significantly lower percentages for urban environments that can act as protective areas in areas with an increase in AWCET than in areas without an increase in AWCET based on the *T*-test.

Urban Environmental Factor	Radius for Spatial Averaging	Average Increase in Relative AWCET for Every 5 Years (1990–2010)		
		Mean (SD) of Areas with Increase	Mean (SD) of Areas without Increase	*p*-Values
Percentage of Vegetation coverage	100 m	**11.4% (16.0%)**	**13.2% (19.9%)**	**<0.05**
	200 m	**12.1% (13.4%)**	**13.1% (15.6%)**	**<0.05**
	300 m	**12.3% (11.6%)**	**13.1% (12.6%)**	**<0.05**
	400 m	**12.5% (10.2%)**	**13.1% (10.7%)**	**<0.05**
	500 m	**12.6% (9.3%)**	**13.3% (9.3%)**	**<0.05**
Percentage of Public Open Space	100 m	9.0% (19.0%)	8.7% (17.0%)	0.34
	200 m	8.6% (13.7%)	8.9% (12.1%)	0.19
	300 m	**8.3% (10.4%)**	**9.1% (9.2%)**	**<0.05**
	400 m	**8.2% (8.1%)**	**9.0% (7.4%)**	**<0.05**
	500 m	**8.1% (6.5%)**	**9.0% (6.3%)**	**<0.05**
Percentage of Road Network	100 m	23.4% (16.6%)	26.7% (16.4%)	<0.05
	200 m	23.1% (12.9%)	26.3% (13.3%)	<0.05
	300 m	22.9% (11.3%)	25.8% (11.7%)	<0.05
	400 m	22.8% (10.2%)	25.4% (10.5%)	<0.05
	500 m	22.7% (9.2%)	25.0% (9.4%)	<0.05
Average Sky View Factor	100 m	0.70 (0.14)	0.63 (0.13)	<0.05
	200 m	0.69 (0.13)	0.64 (0.12)	<0.05
	300 m	0.69 (0.12)	0.64 (0.11)	<0.05
	400 m	0.68 (0.11)	0.65 (0.10)	<0.05
	500 m	0.68 (0.10)	0.65 (0.10)	<0.05

**Table 4:** Comparison of the average increase in AWCET for every five years with areas with poorer/better sustainable planning in the current year, for each type of socioeconomically deprived neighborhood. Bold text indicates results with significantly lower percentages of urban environments that can act as protective areas in areas with an increase in AWCET than in areas without an increase in AWCET based on the *T*-test.

Socioeconomic Deprived Areas	Urban Environmental Factor	Radius for Spatial Averaging	Average Increase in Relative AWCET for Every 5 Years (1990–2010)		
			Mean (SD) of Areas with Increase	Mean (SD) of Areas without Increase	*p*-Values
Areas with higher percentage of senior population (age >= 65)	Percentage of Vegetation coverage	100 m	**17.1% (18.4%)**	**19.7% (23.8%)**	**<0.05**
		200 m	18.7% (14.6%)	19.1% (17.6%)	0.32
		300 m	19.3% (12.1%)	18.9 (13.4%)	0.17
		400 m	19.4% (9.9%)	18.7% (10.6%)	<0.05
		500 m	19.1% (8.5%)	18.5% (8.7%)	<0.05
	Percentage of Public Open Space	100 m	15.0% (25.8%)	13.1% (22.0%)	<0.05
		200 m	14.4% (18.3%)	12.5% (15.0%)	<0.05
		300 m	13.6% (13.0%)	12.2% (10.6%)	<0.05
		400 m	12.5% (9.4%)	11.9% (8.1%)	<0.05
		500 m	11.7% (7.1%)	11.5% (6.7%)	0.35
	Percentage of Road Network	100 m	24.0% (13.7%)	25.3% (13.9%)	<0.05
		200 m	23.7% (10.1%)	25.0% (10.5%)	<0.05
		300 m	23.4% (8.4%)	24.7% (8.4%)	<0.05
		400 m	23.5% (7.3%)	24.5% (6.9%)	<0.05
		500 m	23.6% (6.5%)	24.3% (5.9%)	<0.05
	Average Sky View Factor	100 m	0.64 (0.11)	0.61 (0.10)	<0.05
		200 m	0.64 (0.09)	0.62 (0.09)	<0.05
		300 m	0.65 (0.08)	0.02 (0.08)	<0.05
		400 m	0.64 (0.07)	0.63 (0.07)	<0.05
		500 m	0.64 (0.06)	0.63 (0.06)	<0.05
Areas with higher percentage of children and young adolescent populations (age <= 14)	Percentage of Vegetation coverage	100 m	12.8% (16.5%)	13.3% (21.1%)	0.22
		200 m	13.3% (14.1%)	12.9% (16.7%)	0.33
		300 m	13.4% (12.2%)	12.8% (13.4%)	<0.05
		400 m	13.6% (10.5%)	12.7% (11.1%)	<0.05
		500 m	13.8% (9.4%)	12.8% (9.5%)	<0.05
	Percentage of Public Open Space	100 m	13.8% (23.2%)	10.6% (19.3%)	<0.05
		200 m	12.4% (16.8%)	10.8% (14.1%)	<0.05
		300 m	11.2% (12.1%)	10.9% (10.5%)	0.42
		400 m	10.5% (9.0%)	10.8% (8.0%)	0.27
		500 m	**10.1% (6.8%)**	**10.5% (6.4%)**	**<0.05**
	Percentage of Road Network	100 m	27.7% (15.3%)	30.4% (15.6%)	<0.05
		200 m	27.0% (11.2%)	29.7% (12.6%)	<0.05
		300 m	26.6% (10.0%)	29.0% (11.1%)	<0.05
		400 m	26.1% (9.1%)	28.2% (10.0%)	<0.05
		500 m	25.6% (8.4%)	27.3% (9.1%)	<0.05
	Average Sky View Factor	100 m	0.66 (0.10)	0.61 (0.10)	<0.05
		200 m	0.65 (0.09)	0.62 (0.09)	<0.05
		300 m	0.65 (0.08)	0.63 (0.08)	<0.05
		400 m	0.65 (0.08)	0.63 (0.08)	<0.05
		500 m	0.66 (0.07)	0.64 (0.08)	<0.05
Areas with lower median monthly income from main employment of working population excluding unpaid family workers	Percentage of Vegetation coverage	100 m	**5.8% (9.6%)**	**9.8% (17.6%)**	**<0.05**
		200 m	**7.1% (9.2%)**	**9.8% (13.7%)**	**<0.05**
		300 m	**7.9% (9.0%)**	**10.2% (11.3%)**	**<0.05**
		400 m	**8.5% (8.6%)**	**10.7% (9.9%)**	**<0.05**
		500 m	**9.1% (8.1%)**	**11.2% (8.9%)**	**<0.05**
	Percentage of Public Open Space	100 m	**3.5% (9.7%)**	**7.0% (14.9%)**	**<0.05**
		200 m	**4.2% (7.7%)**	**7.2% (10.0%)**	**<0.05**
		300 m	**5.0% (7.2%)**	**7.3% (7.4%)**	**<0.05**
		400 m	**5.6% (6.3%)**	**7.5% (6.3%)**	**<0.05**
		500 m	**6.1% (5.6%)**	**7.6% (5.7%)**	**<0.05**
	Percentage of Road Network	100 m	24.4% (18.4%)	28.8% (17.9%)	<0.05
		200 m	23.8% (15.5%)	28.5% (15.4%)	<0.05
		300 m	23.4% (14.0%)	28.0% (13.8%)	<0.05
		400 m	23.1% (12.7%)	27.4% (12.5%)	<0.05
		500 m	23.0% (11.4%)	26.7% (11.3%)	<0.05
	Average Sky View Factor	100 m	0.72 (0.16)	0.64 (0.15)	<0.05
		200 m	0.72 (0.15)	0.65 (0.14)	<0.05
		300 m	0.71 (0.14)	0.65 (0.13)	<0.05
		400 m	0.71 (0.13)	0.66 (0.12)	<0.05
		500 m	0.70 (0.12)	0.66 (0.12)	<0.05
Areas with lower percentage of labor force participation rate	Percentage of Vegetation coverage	100 m	**13.5% (15.7%)**	**17.3% (23.3%)**	**<0.05**
		200 m	**15.3% (13.0%)**	**16.7% (17.6%)**	**<0.05**
		300 m	16.3% (11.4%)	16.5% (13.6%)	0.62
		400 m	16.8% (9.9%)	16.4% (10.9%)	0.13
		500 m	16.8% (8.8%)	16.3% (9.0%)	<0.05
	Percentage of Public Open Space	100 m	**10.0% (18.1%)**	**12.0% (20.6%)**	**<0.05**
		200 m	**10.4% (12.1%)**	**11.6% (13.8%)**	**<0.05**
		300 m	**10.6% (9.1%)**	**11.2% (9.6%)**	**<0.05**
		400 m	**10.5% (7.2%)**	**10.9% (7.2%)**	**<0.05**
		500 m	**10.3% (5.8%)**	**10.7% (6.0%)**	**<0.05**
	Percentage of Road Network	100 m	26.8% (13.9%)	27.8% (14.8%)	<0.05
		200 m	26.2% (10.6%)	27.5% (11.5%)	<0.05
		300 m	25.8% (9.3%)	26.9% (9.6%)	<0.05
		400 m	25.5% (8.2%)	26.5% (8.2%)	<0.05
		500 m	25.3% (7.3%)	25.9% (7.1%)	<0.05
	Average Sky View Factor	100 m	0.63 (0.10)	0.61 (0.10)	<0.05
		200 m	0.63 (0.09)	0.61 (0.08)	<0.05
		300 m	0.63 (0.07)	0.62 (0.07)	<0.05
		400 m	0.63 (0.06)	0.62 (0.07)	<0.05
		500 m	0.63 (0.06)	0.63 (0.06)	0.51
